# LeanCOD: Real-Time Small Camouflaged Object Detection on Edge Devices

**DOI:** 10.3390/s26144354

**Published:** 2026-07-09

**Authors:** Youngjin Kim, Dong He, Young Hoo Cho

**Affiliations:** Research and Development Center, dSPECTER, Seongnam 13449, Republic of Korea; youngjin@dspecter.com (Y.K.); donghe@dspecter.com (D.H.)

**Keywords:** camouflaged object detection, small-object detection, lightweight decoder, real-time inference, edge deployment

## Abstract

Camouflaged object detection (COD) methods suffer severe degradation on small camouflaged objects and remain incapable of real-time inference on edge devices. We propose LeanCOD, a framework that pairs a strong foundation-model encoder with a lightweight decoder to enable high-resolution inference. A size-aware composite loss further strengthens supervision on small camouflaged objects. Our size-wise experiments reveal that the 0–1% extra-small-object regime is the major performance bottleneck for existing COD methods. LeanCOD achieves an $S_{\alpha }$ of 0.915 on COD10K at a 576×576 resolution, outperforming competing methods at equal or lower resolutions. Deployed with TensorRT FP16 on an NVIDIA Jetson AGX Orin, LeanCOD runs at 31.6 FPS while maintaining an $S_{\alpha }$ of 0.908 at a 576×576 resolution, exceeding the 30 FPS real-time threshold.

## 1. Introduction

Camouflaged object detection (COD) segments targets that visually blend into their surroundings through color and texture mimicry [1]. The task finds diverse applications in wildlife monitoring, medical image analysis, and autonomous surveillance. Unlike salient object detection (SOD), where high foreground–background contrast provides a strong prior, COD must resolve fine boundary ambiguity between targets and their surroundings.

This work focuses on single-image region-level binary segmentation under the standard COD benchmark setting; instance-level detection and video COD are beyond the present scope.

Recent COD advances have been driven primarily by increasingly complex decoder designs: specialized attention mechanisms [2], frequency decomposition [3], graph-based interaction [4], and multi-stage refinement pipelines [5,6]. Despite improvements in aggregate performance, the rising complexity overhead presents two barriers to practical deployment, which has received limited attention by existing COD methods.

**Figure 1:**
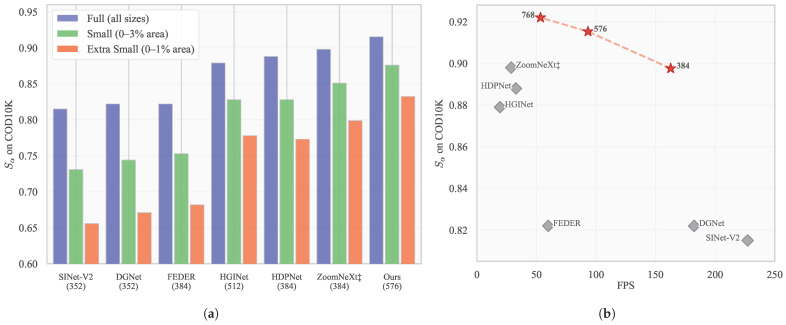
Performance comparison of LeanCOD with existing COD methods on COD10K. (**a**) Performance across small-object regimes. Small and extra-small objects exhibit larger degradation than the full test set, while LeanCOD reduces this degradation. Parenthesized numbers denote input resolutions. (**b**) Performance–FPS comparison on an RTX 4090. Gray points denote existing methods, and red stars denote LeanCOD at 384, 576, and 768 input resolutions. LeanCOD improves accuracy with increasing resolution while maintaining competitive throughput, showing the trade-off between accuracy and throughput. ^‡^ Tri-scale inference.

First, small camouflaged objects produce limited feature-map activations, making accurate localization and boundary delineation challenging. We conduct a size-wise evaluation and reveal that performance drops significantly in small-object regimes, as shown in Figure 1a. This degradation is often obscured by standard aggregate metrics. Second, computational efficiency has received limited attention in the COD community [7]. Although a concurrent study [8] reports benchmarks on embedded platforms, it does not reach the 30 FPS threshold commonly cited for real-time inference.

These two challenges are intrinsically coupled. Higher input resolution is essential to preserve fine detail of small objects, yet the complexity of modern COD decoders precludes real-time inference on resource-constrained platforms. These limitations motivate an alternative design strategy: rather than engineering a more efficient complex decoder, we investigate whether decoder complexity can be radically reduced and the saved computation redirected to input resolution scaling.

We propose LeanCOD, a framework built on the principle of reallocating computation from decoder complexity to input resolution. Because COD relies heavily on semantic context to resolve visual ambiguity, this strategy relies on an encoder that provides sufficiently expressive features, allowing the decoder to remain lightweight without compensating through additional modules. To this end, we systematically benchmark 16 backbones, including CNNs, hierarchical vision transformers, and vision foundation models, and adopt DINOv3-ConvNeXt-Base [9] as the encoder for its accuracy–efficiency trade-off. The resulting decoder uses only standard operations, namely convolutions, element-wise addition, and bilinear upsampling. Furthermore, we supervise training with a size-aware composite loss designed to counteract the severe foreground–background imbalance and the training bias toward large objects.

As shown in Figure 1b, LeanCOD achieves competitive COD accuracy $S_{\alpha }$ on COD10K while maintaining high inference throughput. Furthermore, our TensorRT FP16 deployment reaches 31.6 FPS on an NVIDIA Jetson AGX Orin, exceeding the 30 FPS real-time threshold while maintaining competitive accuracy. To the best of our knowledge, among the published COD methods compared, no other method has simultaneously achieved competitive accuracy and real-time throughput on a Jetson-class edge device.

This work makes the following threefold contributions:

- We propose LeanCOD, a COD framework that exploits the strong representational capacity of a foundation-model encoder to simplify the decoder and redirect the saved computation to high-resolution inference. LeanCOD achieves strong overall accuracy, reaching an $S_{\alpha }$ of 0.915 on COD10K at a 576×576 resolution.
- We introduce a size-aware evaluation protocol that reveals where performance degradation occurs across object scales. It identifies the 0–1% extra-small-object regime as the most challenging case. This analysis motivates our size-aware composite loss and resolution-scaling strategy.
- We report the real-time COD edge deployment on an NVIDIA Jetson AGX Orin using TensorRT FP16. The deployed model reaches 31.6 FPS while maintaining an $S_{\alpha }$ of 0.908, exceeding the 30 FPS real-time threshold.

## 2. Related Work

### 2.1. Camouflaged Object Detection

The introduction of COD10K [1] established large-scale camouflaged object detection as a distinct task, with SINet and its successor SINet-V2 [5] setting early benchmarks through search-and-identification architectures. Subsequent methods have diversified along several design philosophies: CamoFormer [2] separates foreground and background regions through masked separable attention; FEDER [3] decomposes features into frequency bands to reconstruct edges; and HGINet [4] pairs dynamic token clustering with graph-based bidirectional interaction. Furthermore, ZoomNeXt [6], FSPNet [10], and HDPNet [11] refine predictions through pyramid or hierarchical feature propagation, while DGNet [12] introduces gradient-supervised context–texture learning within an efficient dual-branch framework. A recent COD survey [7] identifies multi-scale context, attention, and coarse-to-fine refinement as prevailing design paradigms. However, this evolution has led to excessively heavy decoder architectures, where the pursuit of performance comes at the expense of inference throughput and deployment feasibility.

More recently, vision foundation models (VFMs) have been introduced into COD. EVP [13] demonstrates explicit visual prompting for adapting frozen pretrained transformer backbones to foreground segmentation tasks, including COD. SAM [14]-based pipelines [15] adapt the foundation backbone through prompts or adapter modules. While these works demonstrate the efficacy of VFM-based COD, they primarily focus on encoder adaptation and overlook the potential for architectural simplification in the decoder. LeanCOD investigates this under-explored trade-off, advocating for a substantial reduction in decoder complexity.

### 2.2. Small Camouflaged Object Detection

Standard COD evaluation typically reports aggregate metrics over objects of all sizes, which masks performance degradation on small targets. Li et al. [16] show that SOD metrics are systematically biased toward large objects; Cheng et al. [17] report steep accuracy drops across four size bins in general small-object detection. Within COD, Lv et al. [18] move toward size-aware evaluation by treating objects smaller than 3% of the image area as small-structure targets. However, binary classification offers only coarse-scale characterization and cannot reveal how performance changes across different object-size ranges. Therefore, we instead adopt a six-bin size-aware decomposition, with particular emphasis on the 0–1% extra-small regime that prior work does not isolate from the broader small-object category.

### 2.3. Efficient COD and Edge Deployment

Real-time edge deployment remains largely unexplored in COD [7]. TinyCOD [19] pairs a compact TinyNet-a backbone with an adjacent-scale fusion decoder, and DGNet-S [12] offers a lightweight variant of DGNet. However, neither reports benchmarks on edge devices, and both show lower aggregate accuracy than recent high-performing COD baselines. LiteCOD [8] combines MobileViT-S with holistic local–global feature fusion and reports approximately 20 FPS on an NVIDIA Jetson AGX Orin at a 512×512 resolution, representing one of the earliest published edge-device speed results in the COD literature. However, this measurement was conducted without deployment-oriented inference optimization, and the potential speed gains from such optimization remain unquantified. LiteCOD operates in a complementary design regime to ours: it pairs a compact backbone with additional task-specific fusion modules, whereas our approach relies on a foundation backbone with a lightweight decoder.

### 2.4. Asymmetric Encoder–Decoder Design

In the broader segmentation literature, DeepLabv3+ [20] pairs an encoder augmented with atrous spatial pyramid pooling with a lightweight refinement decoder. SegFormer [21] reduces the decoder to an MLP-only head that relies entirely on a hierarchical transformer encoder for representational capacity. RTFormer [22] achieves real-time segmentation throughput through GPU-friendly attention with a lightweight segmentation head. These works show that a capable encoder paired with a minimal decoder yields competitive accuracy at reduced cost; however, whether this principle extends to COD, where minimal foreground–background contrast demands richer encoder features, has not been explored. LeanCOD investigates this question, reallocating the saved decoder computation to high-resolution inference for small camouflaged objects.

## 3. Methodology

### 3.1. Framework Overview

**Figure 2:**
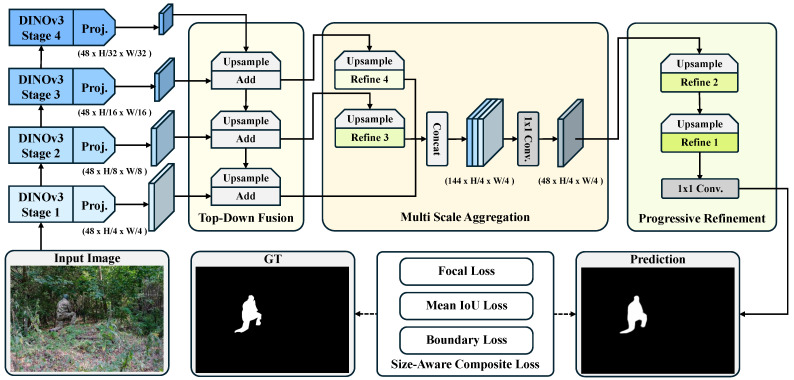
Overall framework of LeanCOD. The DINOv3-ConvNeXt encoder (left) extracts four feature maps with resolutions ranging from H/4 to H/32. These features are projected to a 48-channel dimension. The top-down fusion module integrates semantic context from deep to shallow scales using bilinear upsampling and element-wise addition. The three fused maps are concatenated at the H/4 resolution and compressed by a 1 × 1 convolution in the aggregation block. Finally, the refinement stage progressively restores the spatial resolution to *H* × *W* to generate the prediction map, supervised by the size-aware composite loss. Solid arrows denote the forward data flow, and dashed arrows indicate supervision signals from the loss function. Color gradients illustrate the progressive processing within each stage.

The overall framework of LeanCOD is illustrated in Figure 2. We adopt an asymmetric encoder–decoder paradigm, leveraging a lightweight decoder to concentrate computational resources on enhancing representation learning within the encoder stage. This design is motivated by the observation that high-resolution inputs are crucial for retaining spatial details of small camouflaged objects. Consequently, the encoder must generate rich semantic features to tackle camouflage-induced visual ambiguity. Specifically, we employ DINOv3-ConvNeXt-Base [9] as the encoder, which generates four hierarchical feature maps $\left\{f_{1},f_{2},f_{3},f_{4}\right\}$ at strides {4,8,16,32}. These multi-level features are then fed into a lightweight decoder, which integrates cross-scale cues and predicts a full-resolution camouflage map. To enhance the detection of small targets, we supervise the training with a size-aware composite loss.

### 3.2. Lightweight Decoder

The decoder consists of four stages: channel projection, top-down fusion, multi-scale aggregation, and progressive refinement. By leveraging basic components such as standard convolutions and bilinear upsampling, the decoder efficiently transforms multi-level features into a high-resolution camouflage map. This architecture achieves accurate predictions while maintaining a low computational cost.

#### 3.2.1. Channel Projection

To align channel dimensions, each hierarchical feature map is mapped to 48 channels through a 1 × 1 convolution, complemented by GroupNorm and GELU:

(1) $$f_{i}^{\mathrm{proj}}=\mathrm{GELU}(\mathrm{GN}({\mathrm{Conv}}_{1\times 1}\left(f_{i}\right))),\;i\in \left\{1,2,3,4\right\},$$

where $f_{i}$ denotes the feature map from the *i*-th DINOv3-ConvNeXt stage.

#### 3.2.2. Top-Down Fusion

Given the rich multi-scale representations provided by the encoder, we use simple element-wise addition to propagate high-level contextual information from deeper to shallower levels. Top-down fusion is performed using non-learnable bilinear upsampling followed by element-wise addition:

(2) $$\begin{array}{cc} f_{3}^{\mathrm{fus}} & =f_{3}^{\mathrm{proj}}+{\mathrm{Up}}_{4\to 3}\left(f_{4}^{\mathrm{proj}}\right),\\ f_{2}^{\mathrm{fus}} & =f_{2}^{\mathrm{proj}}+{\mathrm{Up}}_{3\to 2}\left(f_{3}^{\mathrm{fus}}\right),\\ f_{1}^{\mathrm{fus}} & =f_{1}^{\mathrm{proj}}+{\mathrm{Up}}_{2\to 1}\left(f_{2}^{\mathrm{fus}}\right), \end{array}$$

where ${\mathrm{Up}}_{j\to i}\left(\cdot \right)$ denotes bilinear interpolation to the spatial resolution of $f_{i}^{\mathrm{proj}}$, and $f_{i}^{\mathrm{fus}}$ denotes the fused feature at stage *i*.

#### 3.2.3. Multi-Scale Aggregation

Given the multi-scale fused features $\left\{f_{1}^{\mathrm{fus}},f_{2}^{\mathrm{fus}},f_{3}^{\mathrm{fus}}\right\}$, we aggregate them at the resolution of $f_{1}^{\mathrm{fus}}$. Specifically, $f_{2}^{\mathrm{fus}}$ and $f_{3}^{\mathrm{fus}}$ are upsampled and refined by a 3 × 3 convolution followed by GroupNorm and GELU:

(3) $$f_{i}^{\mathrm{ref}}=\mathrm{GELU}(\mathrm{GN}({\mathrm{Conv}}_{3\times 3}\;({\mathrm{Up}}_{i\to 1}\left(f_{i}^{\mathrm{fus}}\right)))),\;i\in \left\{2,3\right\}.$$

The three features are concatenated channel-wise and further integrated via a 1 × 1 convolution followed by GroupNorm, GELU, and spatial dropout with a dropout rate of 0.1:

(4) $$f^{\mathrm{agg}}=\mathrm{Dropout}(\mathrm{GELU}(\mathrm{GN}({\mathrm{Conv}}_{1\times 1}([f_{1}^{\mathrm{fus}};\;f_{2}^{\mathrm{ref}};\;f_{3}^{\mathrm{ref}}])))).$$

In contrast to element-wise addition, we concatenate features from different scales to preserve scale-specific information. This enables the subsequent 1 × 1 convolution to learn adaptive weights across scales, effectively capturing the advantages of multi-scale aggregation without the computational overhead of attention-based fusion.

#### 3.2.4. Progressive Refinement

The aggregated feature $f^{\mathrm{agg}}$ is progressively upsampled to full resolution through two refinement stages. Each stage consists of two bilinear upsamplings followed by a 3 × 3 convolution, GroupNorm, and GELU:

(5) $$\begin{array}{cc} g_{1} & =\mathrm{GELU}(\mathrm{GN}({\mathrm{Conv}}_{3\times 3}\;({\mathrm{Up}}_{2\times }\left(f^{\mathrm{agg}}\right)))),\\ g_{2} & =\mathrm{GELU}(\mathrm{GN}({\mathrm{Conv}}_{3\times 3}\;({\mathrm{Up}}_{2\times }\left(g_{1}\right)))). \end{array}$$

Splitting the fourfold bilinear upsamplings into two steps allows each refinement block to correct aliasing and boundary artifacts at an intermediate resolution before final prediction. The second refinement block reduces the channel capacity from 48 to 24, gradually condensing the representation before the final prediction. Finally, a 1 × 1 convolution maps the 24-channel feature to a logit map $\hat{y}\in R^{1\times H\times W}$.

### 3.3. Feature Flow Visualization

**Figure 3:**
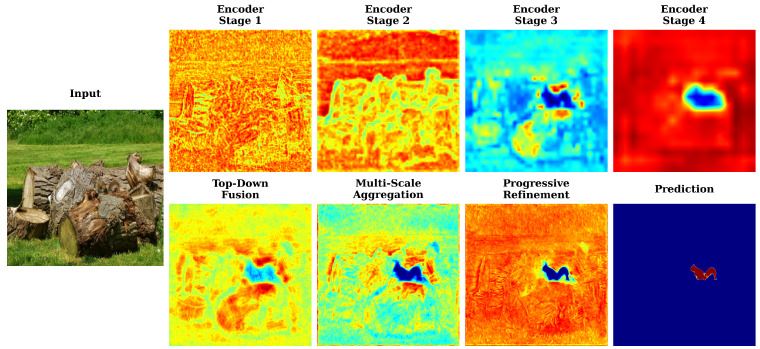
Feature flow visualization of LeanCOD. Early encoder stages capture fine spatial textures, while deeper stages yield robust semantic representations of camouflaged targets. Through top-down fusion, aggregation, and progressive refinement, the decoder propagates the localized target response back to higher spatial resolutions and produces a compact final prediction. Each panel shows the first principal component of feature activations computed by Eigen-CAM, ranging from blue for low values to red for high values.

Figure 3 presents Eigen-CAM [23] visualizations of representative activations within LeanCOD. While early encoder stages preserve fine-grained spatial structures, they are often distracted by background clutter due to target–background similarity. In contrast, deep stages produce semantically selective activations centered on the target, confirming that DINOv3-ConvNeXt extracts sufficiently discriminative features for camouflage resolution. The decoder effectively transfers these high-level responses to higher resolutions through top-down fusion and progressive refinement. This visualization suggests that a lightweight decoder may already be sufficient when the encoder provides strong semantic representations.

### 3.4. Size-Aware Composite Loss

To mitigate the dominance of background pixels and reduce size-induced training bias, we introduce a size-aware composite loss that combines three terms: focal loss [24] for hard example mining, mean IoU loss for scale-invariant region supervision, and boundary loss for structure refinement. The overall objective is formulated as

(6) $$L=\lambda_{1}\;L_{\mathrm{focal}}+\lambda_{2}\;L_{\mathrm{meanIoU}}+\lambda_{3}\;L_{\mathrm{bdy}},$$

where the weighting factors $\lambda_{1}\;=\;0.35$, $\lambda_{2}\;=\;0.45$, and $\lambda_{3}\;=\;0.20$ are empirically determined via grid search. Each term is detailed below.

**Focal loss.** To address the severe imbalance between foreground and background pixels, focal BCE loss down-weights easy pixels and emphasizes hard pixels:

(7) $$L_{\mathrm{focal}}=-\frac{1}{N}\sum_{i=1}^{N}\alpha_{i}{\left(1-p_{t,i}\right)}^{\gamma }\log\left(p_{t,i}\right),$$

where *N* is the number of pixels, $y_{i}\in \left\{0,1\right\}$ is the ground-truth label of the *i*-th pixel, and $p_{i}=\sigma \left({\hat{y}}_{i}\right)$ is the predicted probability. Here, $p_{t,i}=p_{i}$ if $y_{i}=1$ and $p_{t,i}=1-p_{i}$ otherwise. The class-balancing factor is defined as $\alpha_{i}=\alpha$ for foreground pixels and $\alpha_{i}=1-\alpha$ for background pixels. We set α=0.75 and γ=4.0. Compared with the commonly used setting γ=2.0, we use a larger focusing parameter because foreground pixels occupy only a small fraction of COD images, especially for small camouflaged objects.

**Mean IoU loss.** Mean IoU loss provides scale-agnostic region supervision by averaging batch-wise IoU values rather than accumulating intersection and union over the entire batch.

(8) $$L_{\mathrm{meanIoU}}\;=1-\frac{1}{B}\sum_{b=1}^{B}\frac{\sum_{i=1}^{N}p_{b,i}y_{b,i}}{\sum_{i=1}^{N}p_{b,i}+\sum_{i=1}^{N}y_{b,i}-\sum_{i=1}^{N}p_{b,i}y_{b,i}},$$

where *B* is the batch size, and $p_{b,i}$ and $y_{b,i}$ denote the predicted probability and ground-truth label of the *i*-th pixel in the *b*-th sample, respectively. Because each sample contributes equally regardless of object area, small-object samples are not overwhelmed by large-object samples during batch averaging. This property is particularly beneficial for COD, where camouflaged targets can occupy only a very small portion of the image.

**Boundary loss.** Camouflaged objects often exhibit weak and ambiguous boundaries. To encourage sharper structural delineation, we extract a boundary mask from the ground-truth mask using a fixed 5 × 5 Laplaciankernel:

(9) $$m_{\mathrm{bdy}}=1\left[|K_{5\times 5}*y|>0.1\right],$$

where $K_{5\times 5}$ denotes the fixed Laplacian kernel, ∗ denotes convolution, and $m_{\mathrm{bdy}}$ is a binary mask indicating boundary pixels. BCE is then computed only over the extracted boundary pixels:

(10) $$L_{\mathrm{bdy}}=-\frac{1}{\sum_{i=1}^{N}m_{\mathrm{bdy},i}}\sum_{i=1}^{N}m_{\mathrm{bdy},i}\left[y_{i}\log p_{i}+\left(1-y_{i}\right)\log\left(1-p_{i}\right)\right].$$

This term receives the lowest weight among the three losses as it mainly improves boundary coherence rather than dominating the overall optimization.

## 4. Experiments

### 4.1. Experimental Setup

**Datasets.** The experiments are conducted on four widely used COD benchmarks. CAMO [25] contains 1250 camouflaged images in which objects are concealed in natural scenes. CHAMELEON [26] consists of 76 hand-annotated camouflaged images. COD10K [1], the largest COD benchmark, comprises 5066 camouflaged images, 1934 non-camouflaged images, and 3000 background images across 78 categories; only the camouflaged subset is used in this work. NC4K [27] provides 4121 testing images collected from the internet. Consistent with [1,2,6], the training set is constructed by combining the COD10K training set (3040 images) and the CAMO training set (1000 images). Evaluation is performed on CHAMELEON (76 images), CAMO test (250 images), COD10K test (2026 images), and NC4K (4121 images).

**Metrics.** We adopt four standard metrics for quantitative evaluation: S-measure ($S_{\alpha }$) [28], adaptive E-measure ($E_{\phi }$) [29], weighted F-measure ($F_{\beta }^{\omega }$) [30], and mean absolute error (MAE). For object-size analysis on COD10K, the test set is partitioned into six disjoint subsets according to the object-to-image area ratio, defined as the total nonzero foreground pixel count in the binary ground-truth mask divided by the total pixel count of the image. For images containing multiple camouflaged targets, the foreground areas of all targets are summed, and each image is assigned to exactly one bin based on this aggregate ratio. The six bins are 0–1% (188 images), 1–3% (397 images), 3–5% (296 images), 5–10% (498 images), 10–25% (512 images), and >25% (135 images). Throughout this paper, objects below 3% area are referred to as small objects, while objects with 0–1% area are denoted as extra-small objects.

**Implementation Details.** We implement our model inPyTorch 2.7.1 and conduct training on an NVIDIA RTX 4090 GPU. The encoder is a DINOv3-ConvNeXt-Base (88 M parameters) initialized with pretrained weights [9]. To leverage the strong representational priors of the foundation model while allowing task adaptation, we fine-tune the encoder with a lower learning rate and train the decoder at the base learning rate. The model is trained for 100 epochs using the Adam optimizer [31] with a batch size of 32. The decoder uses a learning rate of $1\times 10^{-4}$, while the pretrained encoder uses $1\times 10^{-5}$. For real-world deployment analysis, we export the model to TensorRT with FP16 precision and measure inference performance on an NVIDIA Jetson AGX Orin.

### 4.2. Comparison with State-of-the-Art Methods

**Quantitative comparison.** Table 1 compares LeanCOD with existing COD methods on four standard benchmarks. The lightweight COD block provides an accuracy-oriented reference with three representative efficient methods: TinyCOD, DGNet-S, and LiteCOD. These methods report lower aggregate accuracy across COD10K, CAMO, and NC4K; for example, on COD10K, their $S_{\alpha }$ values range from 0.810 to 0.852, compared with 0.898 for LeanCOD at 384×384. At an input resolution of 384×384, LeanCOD achieves the highest or tied-highest $S_{\alpha }$ on all four datasets among the methods evaluated at 384×384 or lower resolutions. When evaluated at higher resolutions, LeanCOD maintains consistently strong results across $S_{\alpha }$, $E_{\phi }$, $F_{\beta }^{\omega }$, and MAE. These results indicate that the proposed lightweight design effectively benefits from high-resolution inference while preserving competitive overall accuracy.

**Figure 4:**
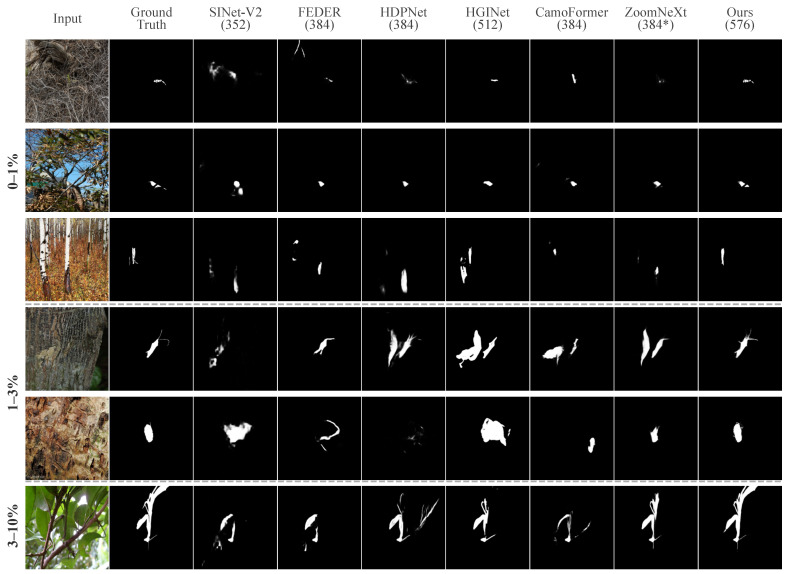
Qualitative comparison on COD10K samples grouped by object area ratio. Rows are organized into three size groups (0–1%, 1–3%, and 3–10%), separated by dashed lines. Columns: input, ground truth, and predictions from six methods. Parenthesized numbers denote input resolution. * denotes tri-scale inference.

**Qualitative comparison.** Figure 4 presents a visual comparison of predictions across three object-size regimes. For extra-small objects (0–1%), the existing methods often exhibit fragmented or weak target responses due to the scarcity of foreground evidence. In contrast, LeanCOD generates spatially concentrated and complete masks. In the 1–3% and 3–10% regimes, LeanCOD produces sharper boundaries and higher structural integrity, particularly in cases of high appearance similarity between target and background. These results demonstrate that high-resolution inference, facilitated by our lightweight decoder, effectively preserves fine-grained spatial details.

**Size-wise experiments.** To evaluate the robustness of LeanCOD across various scales, we extend the binary categorization [18] into a size-aware six-bin decomposition. We define $\Delta =S_{\alpha }\left(0–1\%\right)-S_{\alpha }\left(\mathrm{Full}\right)$ as the performance gap to quantify a model’s sensitivity to extreme scale variations. A smaller |Δ| indicates that the model maintains consistent performance even when foreground evidence is minimal. Table 2 and Table 3 report the size-wise $S_{\alpha }$ and $F_{\beta }^{\omega }$ results, respectively. These experiments yield two key observations:

First, the extra-small regime is most challenging. Performance exhibits a non-linear decay as object size decreases. For objects larger than 3%, the average $S_{\alpha }$ drop between adjacent bins is relatively mild (∼0.020). However, this degradation accelerates sharply in the extra-small regime, with the drop increasing to 0.040 from 3–5% to 1–3% and a staggering 0.091 from 1–3% to 0–1%. This confirms that the 0–1% regime is the primary bottleneck for current COD baselines.

**Figure 5:**
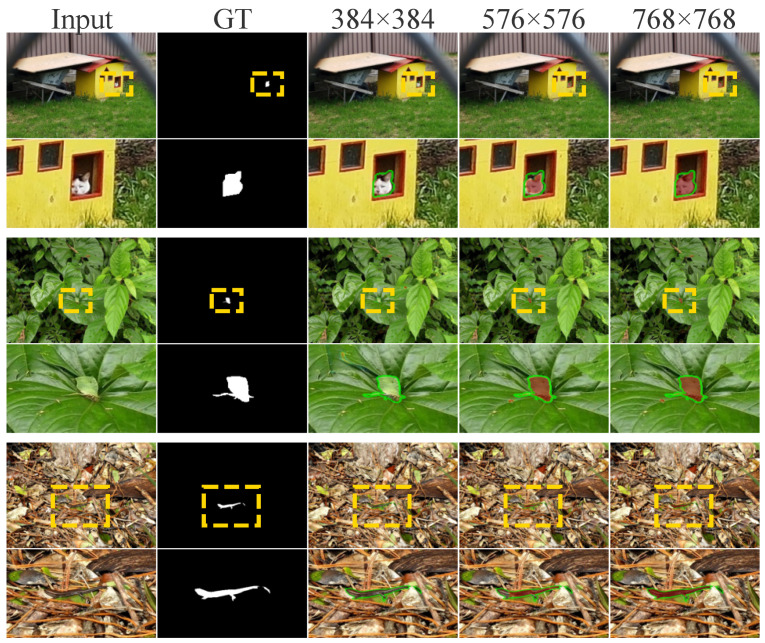
Resolution scaling on three extra-small-object samples (0–1% area) from COD10K. Each example presents the full image, zoomed crop, prediction overlay, and ground-truth boundary in green. Segmentation quality improves progressively as the input resolution increases from 384 to 768. Yellow dashed boxes indicate the zoomed region of interest, and green contours denote the ground-truth boundary.

Second, the resolution-scaling rows of LeanCOD show that increasing input resolution alleviates small-object degradation within the same design. As the input resolution increases, both $S_{\alpha }$ and $F_{\beta }^{\omega }$ improve in the 0–1% bin, and the gap between full-image and extra-small performance decreases. This trend suggests that higher-resolution inputs can help to preserve discriminative spatial structures for extra-small objects, which is further qualitatively validated by the boundary precision improvements in Figure 5.

**Performance–FPS trade-off.** Table 4 compares performance, throughput, and peak memory on the COD10K test set under identical RTX 4090 settings. To examine whether the accuracy gain at higher resolutions is specific to our design, we select three baselines for cross-resolution comparison: SINet-V2 and DGNet as high-throughput representatives and ZoomNeXt as a high-accuracy representative. SINet-V2 and DGNet are retrained at 576 and 768 using each model’s original optimizer, loss, and augmentation (^★^); ZoomNeXt(B4) is evaluated via its tri-scale inference protocol at each nominal resolution (^‡^).

**Figure 6:**
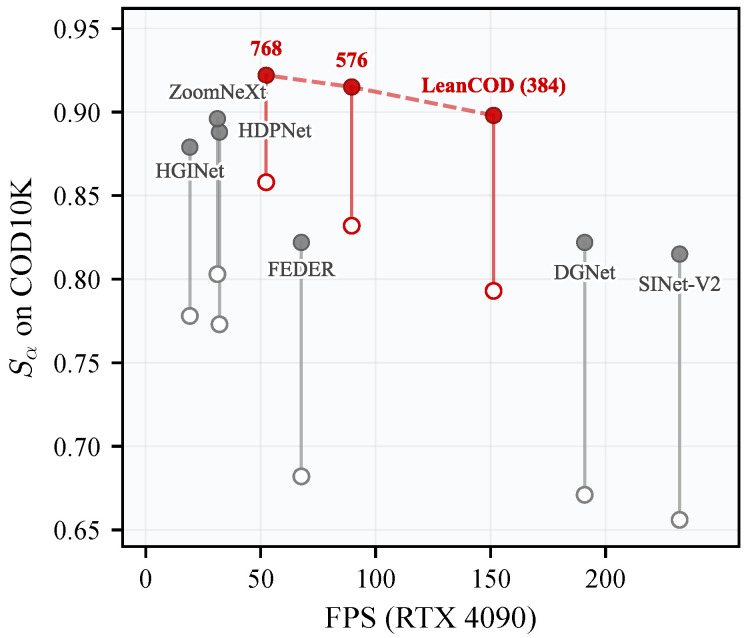
Speed vs. overall and extra-small $S_{\alpha }$ on RTX 4090 (COD10K). Filled: overall $S_{\alpha }$; hollow: extra-small (0–1%) $S_{\alpha }$; vertical lines: degradation gap. Red points denote LeanCOD at 384, 576, and 768 input resolutions.

At 576, SINet-V2^★^ and DGNet^★^ improve by +0.025 and +0.040 in $S_{\alpha }$ over their native 352 results, confirming that training-resolution matching contributes to accuracy. At the same resolution, LeanCOD obtains $S_{\alpha }$ 0.915, compared with 0.840 (SINet-V2^★^) and 0.862 (DGNet^★^). ZoomNeXt uses tri-scale inference, so its effective maximum input at nominal 384 is already 576; at nominal 768, the largest scale reaches 1152, and $S_{\alpha }$ drops to 0.889. These results indicate that resolution scalability varies across architectures. Figure 6 visualizes the speed–accuracy trade-off across all compared methods.

### 4.3. Synthetic Adverse-Condition Evaluation

Following the common-corruption robustness benchmarking practice [34], we evaluate LeanCOD under three synthetic input corruptions: fog, rain, and low light. The corrupted inputs are derived from COD10K test images while reusing the original ground-truth masks; no training, fine-tuning, or inference-time adaptation is applied. LeanCOD is evaluated at a 576×576 input resolution.

**Figure 7:**
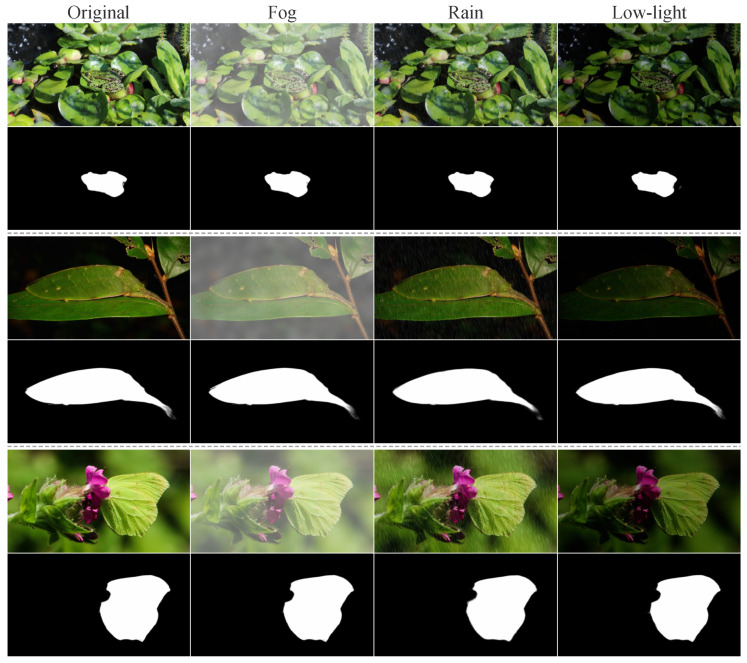
Qualitative results under synthetic adverse conditions on COD10K. Each row pair shows the input image and the predicted mask from LeanCOD. Columns denote original, fog, rain, and low-light conditions. Object location and overall shape are preserved across conditions, although some rain and low-light inputs show weaker interior responses.

Table 5 reports the results, where $\Delta S_{\alpha }$ denotes the change from the original condition. The largest degradation is $\Delta S_{\alpha }=-0.0245$ (rain on full COD10K), while fog and low light produce smaller changes. In the extra-small 0–1% subset, $|\Delta S_{\alpha }|$ remains within 0.0250 for all three corruptions. Figure 7 confirms that object location and overall shape are well preserved across conditions.

### 4.4. Ablation Study

#### 4.4.1. Backbone Ablation

Table 6 benchmarks 16 backbone architectures to investigate their impact on camouflage resolution. The results reveal that backbone selection is decisive for detecting extra-small targets. Under similar parameter scales, foundation backbones exhibit superior semantic robustness over hierarchical ViTs. Notably, this advantage is more pronounced in the extra-small regime: compared with PVTv2-B4, DINOv3-ViT-B improves $S_{\alpha }$ by +0.016 on the full test set, a gain that nearly doubles to +0.029 on the 0–1% bin.

We select DINOv3-ConvNeXt-Base as our default backbone for two reasons. First, its hierarchical architecture naturally provides multi-scale feature maps, which are essential for our fusion-based decoder. Second, it strikes an optimal balance between accuracy and efficiency; while matching the performance of DINOv3-ViT-B, it requires 19% fewer GFLOPs and achieves a 12% higher throughput.

#### 4.4.2. Decoder Ablation

We investigate the necessity of decoder components, namely multi-scale aggregation, progressive refinement, and top-down fusion. Table 7 shows that all decoder variants remain within 0.004 $S_{\alpha }$ of the LeanCOD decoder in overall performance, suggesting that the strong foundation backbone serves as the primary source of representation in this setting. Compared with the FPN baseline [35], the LeanCOD decoder yields +0.003 in overall $S_{\alpha }$ and +0.011 in extra-small $S_{\alpha }$. These margins indicate that the LeanCOD decoder is a low-cost aggregation and refinement choice that preserves strong encoder representations while providing a small complementary benefit in the extra-small regime. Replacing additive top-down fusion with a learned sigmoid gate does not improve performance, suggesting that parameter-free additive fusion is adequate when the encoder already supplies rich contextual features.

Table 8 reports the LeanCOD decoder’s parameter count and computational cost as fractions of the full network, alongside two simpler controls. The LeanCOD decoder accounts for 0.1464% of the total parameters and 5.80% of the total GFLOPs. The S0 linear head applies a 1 × 1 classifier and bilinear upsampling directly to the H/4 encoder feature, reaching 0.477 overall $S_{\alpha }$. The S1 single-upsample multi-scale head omits progressive refinement and recovers 0.895 overall $S_{\alpha }$ with approximately 18% of the LeanCOD decoder’s computation. The LeanCOD decoder provides +0.003 overall $S_{\alpha }$ and +0.011 extra-small $F_{\beta }^{\omega }$ over S1, indicating that progressive refinement contributes primarily to extra-small-object recovery at negligible additional cost.

Table 9 compares bilinear interpolation with DySample, a representative learnable upsampling operator, under the same 100-epoch independent training setting. Replacing only the top-down path with DySample (TD) yields $S_{\alpha }$ 0.897, marginally below bilinear (0.898), while reducing throughput from 158.44 to 149.71 FPS. Replacing all the decoder upsampling paths (All) gives $S_{\alpha }$ 0.899 and improves the extra-small $F_{\beta }^{\omega }$ from 0.587 to 0.602, but throughput drops to 138.10 FPS. These results indicate that learnable upsampling does not provide a meaningful accuracy advantage in this setting while incurring a clear throughput cost. We therefore retain bilinear interpolation in the top-down path.

#### 4.4.3. Loss Ablation

We evaluate each loss component by incrementally composing the size-aware composite loss. Table 10 shows that adding mean IoU to focal BCE yields the largest improvement. Focal BCE addresses pixel-level imbalance, whereas mean IoU introduces sample-wise averaging, allowing each training image to contribute equally regardless of object size. Boundary loss further improves both the overall test set and the extra-small-object subset, although with a smaller gain, reflecting its role in structural refinement.

### 4.5. Edge Deployment

To evaluate practical edge deployment, we export LeanCOD to ONNX with opset 18 and convert it to a TensorRT 10.3 FP16 engine. The experiments are conducted on an NVIDIA Jetson AGX Orin. Table 11 reports the measured accuracy of TensorRT inference and the deployment gap relative to PyTorch evaluation.

At a 576×576 resolution, LeanCOD reaches 31.6 FPS while maintaining an $S_{\alpha }$ of 0.908 on COD10K. This exceeds the commonly used 30 FPS threshold for real-time inference and remains competitive with state-of-the-art COD methods in Table 1. Although the FP16 deployment gap increases with resolution, the 576×576 setting provides the best balance between accuracy and edge throughput.

**Low-power operating analysis.** Edge deployment commonly operates under low-power conditions. We therefore measure LeanCOD across three nvpmodel power modes (15 W, 30 W, and 50 W) on the Jetson AGX Orin at input resolutions of 384×384, 576×576, and 768×768. For each configuration, Table 12 reports throughput (FPS), GPU–SoC power, per-inference energy, and energy efficiency (FPS/W). Power is measured via the tegrastats utility as the VDD_GPU_SOC value, which represents the power supplied to the GPU and SoC compute units and thus reflects inference-relevant power isolated from peripheral components such as I/O controllers. Per-inference energy is the product of the measured power and the engine-only latency.

The table reveals two trends. First, throughput increases substantially with the power budget: raising the mode from 15 W to 50 W at 576×576 increases throughput roughly fourfold, from 7.6 FPS to 31.6 FPS. Consequently, the 30 FPS real-time threshold is met only at 50 W with 384×384 and 576×576 resolutions; 768×768 remains below 30 FPS in all modes. Second, raising the power budget does not proportionally increase per-inference energy because the higher power draw is offset by shorter latency. The 30 W and 50 W modes achieve similar efficiency and both outperform 15 W; at 576×576 the 50 W mode is the most efficient at 1.57 FPS/W. Based on the accuracy in Table 11 and the energy efficiency in Table 12, we adopt 50 W at a 576×576 resolution as the default deployment setting.

## 5. Conclusions

This paper presents LeanCOD, a framework that pairs a foundation encoder with a lightweight decoder for single-image binary COD. Our extensive experiments suggest that, for extra-small camouflaged object detection, allocating computation to spatial resolution is consistently more effective than increasing decoder complexity. Through a size-aware analysis, we identified the 0–1% extra-small-object regime as a major performance bottleneck, which motivated the proposed size-aware loss and resolution-scaling strategy. Evaluated on an NVIDIA Jetson AGX Orin, LeanCOD achieves 31.6 FPS with an $S_{\alpha }$ of 0.908, demonstrating its potential for real-time edge deployment. More broadly, our findings suggest that future COD research may benefit more from compute-efficient high-resolution inference than from increasingly sophisticated decoder engineering. Future work includes extending this approach to instance-level small camouflaged object detection and video COD. The adverse-condition analysis in this work focuses on evaluation-only synthetic COD10K corruptions; evaluation on real fog, rain, and low-light COD data with dense segmentation annotations remains an important future extension.

## Figures and Tables

**Table 1:** Comparison with state-of-the-art methods on four COD benchmarks. ^†^ Results are reported by the original papers. ^§^ Results are evaluated from official weights using our evaluation code. ^†§^ Results are paper-reported for CAMO/COD10K/NC4K and evaluated by us for CHAMELEON. ^‡^ Tri-scale inference. ^†‡^ Results are reported by the original paper using tri-scale inference. The first three methods are lightweight baselines listed as an efficiency-oriented reference. The LeanCOD rows at 576 and 768 are listed as a resolution-scaling extension and are not used for rank marking. The arrows indicate whether higher (↑) or lower (↓) values are better. – indicates that the result is not reported in the original paper. Bold and underlined values indicate the best and second-best results, respectively.

Method	Venue	Res.	CHAMELEON (76)				CAMO (250)				COD10K (2026)				NC4K (4121)			
			$S_{\alpha }\;↑$	$E_{\phi }\;↑$	$F_{\beta }^{\omega }\;↑$	MAE ↓	$S_{\alpha }\;↑$	$E_{\phi }\;↑$	$F_{\beta }^{\omega }\;↑$	MAE ↓	$S_{\alpha }\;↑$	$E_{\phi }\;↑$	$F_{\beta }^{\omega }\;↑$	MAE ↓	$S_{\alpha }\;↑$	$E_{\phi }\;↑$	$F_{\beta }^{\omega }\;↑$	MAE ↓
TinyCOD ^†^ [19]	ICASSP’23	384	0.887	0.931	0.814	0.030	0.822	0.890	0.752	0.066	0.811	0.877	0.678	0.036	0.843	0.903	0.766	0.047
DGNet-S ^†^ [12]	MIR’22	352	–	–	–	–	0.826	0.896	0.754	0.063	0.810	0.869	0.672	0.036	0.845	0.902	0.764	0.047
LiteCOD ^†^ [8]	AI’25	512	–	–	–	–	0.841	0.907	0.796	0.056	0.852	0.920	0.765	0.026	0.870	0.926	0.822	0.036
SINet-v2 ^†^ [5]	TPAMI’22	352	0.888	0.942	0.882	0.030	0.820	0.882	0.743	0.070	0.815	0.887	0.680	0.037	0.847	0.903	0.770	0.048
DGNet ^†^ [12]	MIR’22	352	0.891	0.952	0.838	0.024	0.839	0.901	0.769	0.057	0.822	0.903	0.693	0.033	0.857	0.907	0.784	0.042
FEDER ^†^ [3]	CVPR’23	384	0.894	0.947	0.855	0.028	0.807	0.873	0.785	0.069	0.823	0.900	0.740	0.032	0.846	0.905	0.789	0.045
FSPNet ^†^ [10]	CVPR’23	384	0.908	0.965	0.851	0.023	0.856	0.899	0.799	0.050	0.851	0.895	0.735	0.026	0.879	0.915	0.816	0.035
CamoFormer ^§^ [2]	TPAMI’24	384	0.910	0.970	0.866	0.022	0.872	0.931	0.831	0.046	0.869	0.931	0.786	0.023	0.892	0.941	0.847	0.030
ZoomNeXt ^†‡^ [6]	TPAMI’24	384 ^‡^	0.924	**0.975**	0.896	0.018	0.889	0.945	**0.875**	0.041	**0.898**	**0.956**	0.848	0.018	0.903	0.951	0.863	0.028
HGINet ^†^ [4]	TIP’24	512	0.915	0.970	0.889	0.018	0.874	0.937	0.848	0.041	0.882	0.949	0.821	0.019	0.894	0.947	0.865	0.027
FGSA-Net ^†^ [32]	TMM’25	512	0.916	**0.975**	**0.903**	**0.016**	0.889	0.944	0.870	0.036	0.893	0.953	**0.849**	**0.015**	0.903	0.951	**0.883**	**0.023**
HDPNet ^†§^ [11]	WACV’25	384	0.922	0.943	0.861	0.021	0.893	0.934	0.851	0.040	0.888	0.925	0.794	0.020	0.902	0.950	0.850	0.029
ESCNet ^†^ [33]	ICCV’25	416	–	–	–	–	0.871	0.934	0.843	0.044	0.873	0.939	0.804	0.021	0.892	0.941	0.859	0.028
Ours	–	384	**0.925**	0.969	0.871	0.020	**0.904**	**0.949**	0.870	**0.035**	**0.898**	0.944	0.824	0.018	**0.910**	**0.958**	0.870	0.026
Ours (high-res)	–	576	0.935	0.974	0.895	0.018	0.911	0.953	0.881	0.033	0.915	0.956	0.859	0.016	0.915	0.959	0.881	0.026
Ours (high-res)	–	768	0.937	0.971	0.901	0.017	0.913	0.952	0.885	0.033	0.922	0.961	0.874	0.015	0.916	0.959	0.884	0.025

**Table 2:** Size-wise $S_{\alpha }$ performance on the COD10K test set. All external methods use official pretrained weights under identical evaluation. Δ=(0–1%)−Full; smaller |Δ| indicates better robustness to small objects. ^‡^ Tri-scale inference (0.5× + 1× + 1.5× of nominal resolution, e.g., 192 + 384 + 576 for 384). The LeanCOD rows at 576 and 768 are used as a resolution-scaling extension. The arrows indicate whether higher (↑) values are better.

Method	Venue	Res.	$S_{\alpha }$	$S_{\alpha }\;↑$ by Object Area Ratio (%)						$S_{\alpha }$
			**Full**	>**25**	**10–25**	**5–10**	**3–5**	**1–3**	**0–1**	Δ
SINet-v2 [5]	TPAMI’22	352	0.815	0.845	0.873	0.841	0.825	0.767	0.656	−0.159
DGNet [12]	MIR’22	352	0.822	0.855	0.875	0.850	0.823	0.779	0.671	−0.151
FEDER [3]	CVPR’23	384	0.822	0.825	0.866	0.849	0.835	0.786	0.682	−0.140
CamoFormer [2]	TPAMI’24	384	0.869	0.892	0.912	0.894	0.869	0.833	0.748	−0.121
HGINet [4]	TIP’24	512	0.879	0.891	0.912	0.901	0.879	0.851	0.778	−0.101
HDPNet [11]	WACV’25	384	0.888	0.905	0.925	0.911	0.894	0.854	0.773	−0.115
ESCNet [33]	ICCV’25	416	0.874	0.891	0.910	0.897	0.882	0.843	0.753	−0.121
ZoomNeXt ^‡^ [6]	TPAMI’24	384 ^‡^	0.898	0.901	0.927	0.920	0.901	0.875	0.799	−0.099
Ours	–	384	0.898	0.910	0.929	0.919	0.905	0.871	0.793	−0.105
Ours (high-res)	–	576	0.915	0.914	0.937	0.934	0.922	0.897	0.832	−0.083
Ours (high-res)	–	768	0.922	0.917	0.940	0.937	0.928	0.908	0.858	−0.064

**Table 3:** Size-wise $F_{\beta }^{\omega }$ performance on the COD10K test set. $F_{\beta }^{\omega }$ is reported alongside $S_{\alpha }$ as it is particularly sensitive to small-object boundary quality. $\Delta =F_{\beta }^{\omega }\left(0–1\%\right)-F_{\beta }^{\omega }\left(\mathrm{Full}\right)$; smaller |Δ| indicates better robustness to scale variation. ^‡^ Tri-scale inference. The LeanCOD rows at 576 and 768 are used as a resolution-scaling extension. The arrows indicate whether higher (↑) values are better.

Method	Venue	Res.	$F_{\beta }^{\omega }$	$F_{\beta }^{\omega }\;↑$ by Object Area Ratio (%)						$F_{\beta }^{\omega }$
			**Full**	>**25**	**10–25**	**5–10**	**3–5**	**1–3**	**0–1**	Δ
SINet-v2 [5]	TPAMI’22	352	0.680	0.843	0.815	0.728	0.682	0.556	0.326	−0.354
DGNet [12]	MIR’22	352	0.692	0.852	0.821	0.746	0.680	0.573	0.349	−0.343
FEDER [3]	CVPR’23	384	0.715	0.832	0.824	0.768	0.729	0.614	0.384	−0.331
CamoFormer [2]	TPAMI’24	384	0.786	0.896	0.884	0.832	0.781	0.698	0.516	−0.270
HGINet [4]	TIP’24	512	0.815	0.913	0.897	0.854	0.812	0.740	0.579	−0.236
HDPNet [11]	WACV’25	384	0.794	0.909	0.892	0.842	0.798	0.701	0.503	−0.291
ESCNet [33]	ICCV’25	416	0.808	0.913	0.894	0.853	0.818	0.729	0.533	−0.275
ZoomNeXt ^‡^ [6]	TPAMI’24	384 ^‡^	0.827	0.912	0.904	0.868	0.825	0.762	0.585	−0.242
Ours	–	384	0.824	0.915	0.899	0.864	0.831	0.752	0.593	−0.231
Ours (high-res)	–	576	0.859	0.919	0.916	0.892	0.863	0.809	0.675	−0.184
Ours (high-res)	–	768	0.874	0.924	0.921	0.898	0.878	0.834	0.724	−0.150

**Table 4:** Resolution-ordered efficiency and COD10K accuracy comparison on an RTX 4090. We select SINet-V2 and DGNet as high-throughput representatives and ZoomNeXt as a high-accuracy representative for cross-resolution comparison. All measurements use identical settings: batch size 1, 50 warm-up iterations, and 200 timed runs. Peak memory denotes the maximum CUDA memory allocated during inference. ^‡^ indicates tri-scale inference (0.5× + 1.0× + 1.5× of nominal resolution). ^★^ indicates models retrained by us at the listed resolution using the same optimizer, loss, and augmentation as the original papers; these are not the published models and serve as diagnostic baselines. Extra-small denotes the 0–1% object-area-ratio bin of COD10K. The arrows indicate whether higher (↑) or lower (↓) values are better.

		Complexity		RTX 4090		COD10K $S_{\alpha }$	
Method	Res.	Params ↓	GFLOPs ↓	FPS ↑	Peak Mem. (MB) ↓	Full ↑	Extra-Small ↑
SINet-v2 [5]	352	26.98 M	24.3	232.2	136.1	0.815	0.656
DGNet [12]	352	19.22 M	12.8	190.9	132.6	0.822	0.671
ZoomNeXt ^‡^ [6]	384 ^‡^	65.37 M	264.1	31.2	404.9	0.896	0.803
Ours	384	88.43 M	96.5	151.3	420.8	0.898	0.793
SINet-v2 ^★^ [5]	576	26.98 M	65.2	213.9	192.8	0.840	0.701
DGNet ^★^ [12]	576	19.22 M	34.1	178.0	234.6	0.862	0.742
ZoomNeXt ^‡^ [6]	576 ^‡^	65.37 M	686.7	16.1	591.2	0.900	0.831
Ours	576	88.43 M	217.1	89.6	511.2	0.915	0.832
SINet-v2 ^★^ [5]	768	26.98 M	115.9	152.2	258.0	0.843	0.716
DGNet ^★^ [12]	768	19.22 M	60.7	113.9	357.1	0.869	0.750
ZoomNeXt ^‡^ [6]	768 ^‡^	65.37 M	1451.1	8.5	845.8	0.889	0.825
Ours	768	88.43 M	385.9	52.4	640.4	0.922	0.858

**Table 5:** Robustness evaluation under synthetic COD10K corruptions using LeanCOD at a 576×576 input resolution. The fog, rain, and low-light conditions are evaluation-only input variants that reuse the original COD10K ground-truth masks; no adverse-condition images are used for training, fine-tuning, or inference-time adaptation. $\Delta S_{\alpha }$ denotes the change from the original condition; smaller $|\Delta S_{\alpha }|$ indicates better stability under the corruption. Rain produces the largest drop ($\Delta S_{\alpha }=-0.0245$ on full COD10K), while fog and low light yield smaller changes. The arrows indicate whether higher (↑) or lower (↓) values are better. – in the $\Delta S_{\alpha }$ column indicates the reference condition.

Condition	COD10K Full				Extra-Small (0–1%)			
	$S_{\alpha }\;↑$	$F_{\beta }^{\omega }\;↑$	MAE ↓	$\Delta S_{\alpha }$	$S_{\alpha }\;↑$	$F_{\beta }^{\omega }\;↑$	MAE ↓	$\Delta S_{\alpha }$
Original	0.9153	0.8590	0.0158	–	0.8324	0.6746	0.0043	–
Fog	0.9101	0.8519	0.0165	−0.0052	0.8259	0.6605	0.0040	−0.0065
Rain	0.8908	0.8194	0.0207	−0.0245	0.8074	0.6306	0.0058	−0.0250
Low Light	0.9028	0.8403	0.0176	−0.0125	0.8135	0.6430	0.0051	−0.0189

**Table 6:** Backbone comparison on COD10K with the LeanCOD decoder fixed. All models are trained for 100 epochs at 384×384 input resolution. Backbones are grouped by architecture family. Extra-small denotes the 0–1% object-area-ratio subset of COD10K (188 images). CN denotes ConvNeXt. The arrows indicate whether higher (↑) or lower (↓) values are better. Best results are shown in **bold**, and second-best results are underlined.

				COD10K Full		Extra-Small (0–1%)	
Backbone	Params ↓	GFLOPs ↓	FPS ↑	$S_{\alpha }\;↑$	$F_{\beta }^{\omega }\;↑$	$S_{\alpha }\;↑$	$F_{\beta }^{\omega }\;↑$
EfficientNet-B1	**6.62 M**	8.74	236.7	0.819	0.640	0.686	0.335
EfficientNet-B4	17.66 M	14.13	184.2	0.838	0.684	0.708	0.400
ResNet-50	23.77 M	29.93	**458.4**	0.820	0.668	0.690	0.368
ResNet-101	42.76 M	51.75	299.8	0.837	0.685	0.707	0.397
PVTv2-B2	24.98 M	30.88	223.2	0.863	0.748	0.735	0.463
PVTv2-B3	44.86 M	48.60	153.2	0.876	0.769	0.746	0.486
PVTv2-B4	62.17 M	68.51	112.1	0.879	0.776	0.760	0.514
PVTv2-B5	81.57 M	78.69	89.2	0.877	0.775	0.759	0.510
DINOv3-ViT-S	22.12 M	37.71	232.2	0.869	0.741	0.741	0.471
DINOv3-CN-Tiny	28.50 M	32.26	350.5	0.871	0.774	0.745	0.499
DINOv3-ViT-S+	29.22 M	45.93	218.2	0.874	0.753	0.761	0.499
DINOv3-CN-Small	50.13 M	57.11	229.2	0.888	0.805	0.780	0.581
DINOv3-ViT-B	86.59 M	119.17	145.1	0.895	0.799	0.789	0.566
DINOv3-CN-Base	88.43 M	96.48	162.7	0.898	0.824	0.793	0.593
DINOv3-CN-Large	197.45 M	208.51	108.4	0.908	**0.845**	0.813	0.631
DINOv3-ViT-L	304.33 M	392.94	57.8	**0.912**	0.839	**0.822**	**0.644**

**Table 7:** Decoder ablation on COD10K with DINOv3-CN-Base at 384×384 resolution. TDF: top-down fusion; MSA: multi-scale aggregation; PR: progressive refinement. Extra-small denotes the 0–1% object-area-ratio subset of COD10K. ^★^ Learned sigmoid gate replaces parameter-free top-down addition. The arrows indicate whether higher (↑) values are better. ✓ indicates that the corresponding decoder component is included. The selected configuration is shown in **bold**.

				COD10K Full		Extra-Small (0–1%)	
Configuration	TDF	MSA	PR	$S_{\alpha }\;↑$	$F_{\beta }^{\omega }\;↑$	$S_{\alpha }\;↑$	$F_{\beta }^{\omega }\;↑$
FPN Baseline				0.895	0.817	0.782	0.577
LeanCOD w/o MSA	✓		✓	0.894	0.815	0.785	0.574
LeanCOD w/o Progressive	✓	✓		0.896	0.819	0.790	0.583
LeanCOD w/o Top-Down		✓	✓	0.896	0.820	0.789	0.577
LeanCOD + Gated Fusion ^★^	✓	✓	✓	0.896	0.824	0.788	0.584
**LeanCOD Decoder (Ours)**	✓	✓	✓	**0.898**	**0.824**	**0.793**	**0.593**

**Table 8:** Simpler decoder controls and LeanCOD decoder cost with DINOv3-CN-Base at 384×384 input. Decoder parameters and GFLOP ratios are relative to the full network. S0 is a single 1 × 1 linear segmentation head; S1 is a single-upsample multi-scale head. Extra-small denotes the 0–1% object-area-ratio subset of COD10K. The LeanCOD decoder adds 0.1464% of parameters and 5.80% of GFLOPs; the S0 linear head shows that a simple readout is insufficient, while S1 recovers most accuracy at lower cost. The arrows indicate whether higher (↑) values are better. The selected configuration is shown in **bold**.

	Decoder Params		Decoder GFLOPs		COD10K Full		Extra-Small (0–1%)	
Configuration	Count	Ratio	Value	>Ratio	$S_{\alpha }\;↑$	$F_{\beta }^{\omega }\;↑$	$S_{\alpha }\;↑$	$F_{\beta }^{\omega }\;↑$
S0 Linear Head	65	0.0001%	0.0012	0.0013%	0.477	0.157	0.384	0.014
S1 Single-Upsample Multi-Scale Head	98,257	0.1112%	1.0024	1.09%	0.895	0.817	0.787	0.582
**LeanCOD Decoder (Ours)**	**129,481**	**0.1464%**	**5.5951**	**5.80%**	**0.898**	**0.824**	**0.793**	**0.593**

**Table 9:** Top-down upsampling ablation on COD10K with DINOv3-CN-Base at 384×384 resolution. All variants are independently trained for 100 epochs under the same setting, changing only the upsampling operator. DySample (TD) replaces only the top-down path; DySample (All) replaces all decoder upsampling paths. Extra-small denotes the 0–1% object-area-ratio subset of COD10K. The selected method is shown in **bold**. The arrows indicate whether higher (↑) or lower (↓) values are better. Learnable upsampling does not improve accuracy but reduces throughput, supporting the use of bilinear interpolation.

	COD10K Full			Extra-Small (0–1%)			
Top-Down Upsampling	$S_{\alpha }\;↑$	$F_{\beta }^{\omega }\;↑$	MAE ↓	$S_{\alpha }\;↑$	$F_{\beta }^{\omega }\;↑$	MAE ↓	FPS ↑
**Bilinear (Ours)**	**0.898**	0.825	**0.018**	0.791	0.587	**0.006**	**158.44**
DySample (TD)	0.897	0.825	0.018	0.790	0.589	0.006	149.71
DySample (All)	0.899	0.826	0.018	0.797	0.602	0.006	138.10

**Table 10:** Loss ablation on COD10K using DINOv3-CN-Base with the LeanCOD decoder at 384×384 input resolution. Loss components are incrementally added to focal BCE. Extra-small denotes the 0–1% object-area-ratio subset of COD10K (188 images). The arrows indicate whether higher (↑) values are better. ✓ indicates that the corresponding loss component is used. The final configuration is shown in **bold**.

				COD10K Full		Extra-Small (0–1%)	
Configuration	Focal	Mean IoU	Boundary	$S_{\alpha }\;↑$	$F_{\beta }^{\omega }\;↑$	$S_{\alpha }\;↑$	$F_{\beta }^{\omega }\;↑$
Focal Only	✓			0.770	0.420	0.536	0.066
Focal + Mean IoU	✓	✓		0.891	0.831	0.786	0.591
**Full (Ours)**	✓	✓	✓	**0.898**	**0.824**	**0.793**	**0.593**

**Table 11:** Edge deployment on an NVIDIA Jetson AGX Orin using TensorRT FP16. FPS is measured with batch size 1 under 50 W power mode. $\Delta_{S_{\alpha }}$ denotes the gap relative to PyTorch FP32 evaluation in Table 4. Extra-small denotes the 0–1% object-area-ratio bin. The arrows indicate whether higher (↑) values are better.

	Jetson Orin	$S_{\alpha }$ (TRT FP16)		$F_{\beta }^{\omega }$ (TRT FP16)		$\Delta_{S_{\alpha }}$ vs. PyTorch (FP32)	
Res.	FPS ↑	Full	Extra-Small	Full	Extra-Small	Full	Extra-Small
384	56.0	0.897	0.791	0.822	0.590	−0.001	−0.002
576	31.6	0.908	0.822	0.850	0.660	−0.007	−0.010
768	19.4	0.907	0.828	0.859	0.691	−0.015	−0.030

**Table 12:** Inference performance of LeanCOD on an NVIDIA Jetson AGX Orin across nvpmodel power modes (15 W/30 W/50 W). TensorRT FP16, batch size 1, and engine-only FPS. VDD_GPU_SOC is the power rail supplying the GPU and SoC compute units; measuring this rail isolates inference-relevant power from peripheral subsystems. Power is the tegrastats median; energy per inference is power × engine-only latency; energy efficiency is FPS/W. The bolded 50 W/576×576 is the default deployment configuration (see Table 11 for detection accuracy). The arrows indicate whether higher (↑) or lower (↓) values are better. ✓ and ✕ indicate whether the configuration meets or does not meet the 30 FPS real-time threshold, respectively.

Power Mode	Res.	FPS ↑	VDD_GPU_SOC (W)	Energy/inf. (mJ) ↓	FPS/W ↑	Real-Time (≥30 FPS)
15 W	384	15.3	5.63	367.8	2.72	✕
15 W	576	7.6	6.03	795.9	1.26	✕
15 W	768	4.2	6.03	1424.7	0.70	✕
30 W	384	27.6	8.84	320.4	3.12	✕
30 W	576	14.0	9.24	658.7	1.51	✕
30 W	768	8.2	9.65	1173.9	0.85	✕
50 W	384	56.0	18.09	323.0	3.10	✓
**50 W**	**576**	**31.6**	**20.10**	**636.0**	**1.57**	✓
50 W	768	19.4	21.30	1098.0	0.91	✕

## Data Availability

The original contributions presented in this study are included in the article. Further inquiries can be directed to the corresponding author.
